# Comparative genomics of carbapenem resistant and susceptible clinical *Acinetobacter baumannii* reveals lineage-associated mobilization of acquired carbapenemase determinants: an integrative *in silico* genomics approach

**DOI:** 10.3389/fcimb.2026.1886564

**Published:** 2026-08-12

**Authors:** Sara Pearl, Anand Anbarasu

**Affiliations:** Department of Biotechnology, School of Bio Sciences and Technology (SBST), Vellore Institute of Technology (VIT), Vellore, India

**Keywords:** *A. baumannii*, antimicrobial resistance, carbapenemase, horizontal gene transfer, PanGWAS, phylogenomics

## Abstract

**Background:**

Carbapenem resistant *Acinetobacter baumannii* (CRAB) is recognized as one of the most critical priority pathogens by the World Health Organization due to its persistence in nosocomial settings, extensive antimicrobial resistance, and increasing dissemination at the global level. Despite the escalating availability of genomic data, genotype–phenotype integrated studies exploring the genetic determinants associated with carbapenem resistance remain limited.

**Methods:**

In this study, a comprehensive comparative genomics was performed using publicly available 395 clinical *A. baumannii* genomes, comprising of 267 CRAB and 128 carbapenem susceptible *A. baumannii* (CSAB). Comparative genomic analyses included sequence types (STs), virulence factors (VFs), antimicrobial resistance genes (ARGs), and mobile genetic elements (MGEs) characterization. Pangenome-wide association study (PanGWAS) was performed to test the associations between genotypes and carbapenem resistance phenotype.

**Results:**

CRAB genome subset demonstrated higher abundance of ARGs (acquired carbapenemases in particular), plasmids, carbapenem resistance-associated insertion sequences, and integrons than CSAB genomes. PanGWAS identified six positively associated genes (*relE*, *umuC*, *hphA*, *hsmA*, *hphR*, and *fecI*) significantly enriched in CRAB population. Core SNP phylogeny integrated with STs and acquired carbapenemase genes exhibited heterogeneous distribution of resistance genes across lineages, indicating potential role of both clonal dissemination and horizontal gene transfer.

**Conclusion:**

This study provides an overall genomic architecture of CRAB integrating comparative genomics, PanGWAS, and phylogenomics approaches. The findings underscore the complex interplay between ARGs, VFs, and MGEs in the genomic evolution of CRAB, expanding current understanding of CRAB adaptation and may contribute toward enhanced surveillance, antimicrobial stewardship, and exploration of alternative therapeutic targets.

## 1 Introduction

*Acinetobacter baumannii* is a member of the clinically important ‘ESKAPE’ group of pathogens, known for causing opportunistic infections in both healthcare and community settings. It is a Gram-negative, aerobic, pleomorphic coccobacillus that lacks flagella but exhibits twitching motility mediated by type IV pili and surface-associated motility (Whiteway et al., 2022). The World Health Organization (WHO) has classified carbapenem-resistant *A. baumannii* (CRAB) into the critical group in the updated Bacterial Priority Pathogens List (World Health Organization, 2024). This organism has the potential to thrive and persist in diverse environments and is frequently implicated in hospital outbreaks, as well as in infections associated with wars, natural disasters, and both tropical and temperate climates. It causes a wide spectrum of infections, including bloodstream infections (BSI), ventilator-associated pneumonia (VAP), meningitis, urinary tract infections (UTIs), and skin and wound infections (Anitha et al., 2014). *A. baumannii* preferentially colonizes moist tissues such as the mucosa of respiratory, gastrointestinal, and urinary tracts, as well as injured skin. Severe infections lead to bacteremia and septicemia, with inappropriate treatments resulting in mortality. In the nosocomial settings, patients at higher risk include those who are immunocompromised, subjected to surgical procedures and prolonged hospitalization, or reliant on invasive medical devices such as catheters and ventilators (Howard et al., 2012).

The surge in emergence of multidrug-resistant (MDR), extensively drug-resistant (XDR), and pandrug-resistant (PDR) *A. baumannii* strains at the global level poses formidable challenges in the treatment of these infections (Carrasco et al., 2021; Assimakopoulos et al., 2023; Lukovic et al., 2025). Carbapenem resistance, in particular, represents a clinical emergency (Vijayakumar et al., 2024). Outbreaks of CRAB have been reported worldwide, often driven by clonal dissemination of carbapenemase-producing CRAB (CP-CRAB) strains (Müller et al., 2023).

The primary mechanism of carbapenem resistance in *A. baumannii* is the production of carbapenem-hydrolyzing *β*-lactamases. Among them, class D oxacillinases, which are known as carbapenem-hydrolyzing class D *β*-lactamases (CHDLs), are the most predominant determinants (Poirel and Nordmann, 2006; Malathi et al., 2017). CHDLs in *A. baumannii* includes intrinsic OXA-51-like enzymes and acquired OXA-23-like, OXA-58-like, OXA-24/40-like, OXA-235-like, OXA-72-like, OXA-48-like, and OXA-143-like groups of enzymes (Thacharodi et al., 2024). Notably, OXA-23 is the prominent determinant of CRAB globally and is frequently mobilized *via* plasmids, facilitating horizontal gene transfer (HGT). A whole-genome sequencing (WGS)-based study of CRAB strains from 120 patients across four U.S. hospital centers revealed that *bla*_OXA-23_ gene was found to be present in 69% of the isolates and the most frequently detected acquired carbapenemase gene (Iovleva et al., 2022). Zhao et al. reported that presence of the *bla*_OXA-23_ gene was the main driver of the CRAB resistance phenotype among the prevalent CC2 clonal type isolates from the intensive care units (ICUs) in eastern China (Zhao et al., 2019). Class A *β*-lactamases (KPC and GES) and class B metallo-*β*-lactamases (MBLs), including NDM, IMP, VIM, and SIM, are also identified in CRAB strains, as additional enzymatic inactivation strategies in conferring resistance (Thacharodi et al., 2024).

Furthermore, alterations in penicillin-binding proteins (PBPs), the conventional targets of carbapenems, reduces drug binding affinity leading to decrease in the inhibition potential. Modifications in the outer membrane permeability due to loss or reduced expression levels of the membrane proteins (porins) decreases influx of antibiotics. CarO is the carbapenem resistance associated outer membrane protein in *A. baumannii* (Poirel and Nordmann, 2006). Additionally, the overexpression of resistance-nodulation-division (RND) efflux systems, especially AdeABC and AdeIJK, reduces the intracellular carbapenem concentrations (Shi et al., 2024).

While resistance determinants of CRAB strains have been widely studied, the genomic features distinguishing carbapenem-resistant and susceptible isolates remain incompletely characterized. In this study, we performed comparative genomic analysis to investigate a curated dataset comprising of carbapenem-resistant and susceptible genomes from *A. baumannii* clinical sources with available phenotypic data. The study integrates resistome profiling, mobile genetic element (MGE) analysis, genotype-phenotype correlation, and population structure characterization to explore the genomic architecture underlying carbapenem resistance.

## 2 Materials and methods

### 2.1 Genomic data collection and metadata-based filtering

Genomic sequences and associated metadata were retrieved from the PubMLST repository (accessed April 2026) (Jolley et al., 2018). A schematic overview of the dataset filtering is presented in **Supplementary Figure 1**. A total of 13,994 genomes were available in the database for *A. baumannii* for which the corresponding metadata file was downloaded. Genomes specifically corresponding to *A. baumannii* in the species field were retained, resulting in 12,863 genomes and excluding entries belonging to other *Acinetobacter* species. Genome entries lacking collection year information or reported before the year 2000 were filtered out, yielding 11,136 genomes. Further, the dataset was filtered based on the source to ensure clinical relevance. Genomes originating from environmental, food, animal, and other non-clinical sources were excluded, resulting in 2,096 genomes. Exclusion of genomes sourced from culture collections, surveillance isolates, or with incomplete source information produced a dataset comprising 2,007 genomes. Surveillance-associated entries were excluded due to lack of detailed isolation source information to confidently determine clinical or nosocomial origin isolated from human host.

To enable carbapenem resistance-associated comparative genomic analysis, only genomes available with antimicrobial susceptibility testing (AST) or minimum inhibitory concentration (MIC) data for the carbapenem antibiotics (imipenem and meropenem) were retained, resulting in 762 genomes. Genomes were categorized into CRAB and carbapenem-susceptible *A. baumannii* (CSAB) based on the reported AST interpretation for the two carbapenems. CRAB included isolates reported as resistant or intermediate/non-susceptible to at least one of the carbapenems, whereas CSAB included isolates reported as susceptible to both imipenem and meropenem. Intermediate/non-susceptible isolates were classified into the CRAB group because reduced carbapenem susceptibility may reflect partial or emerging resistance trends with potential clinical relevance. To ensure phenotype confidence for downstream comparative analyses, only genomes with available AST data for both carbapenems were retained, while isolates with missing susceptibility information were excluded from the dataset. This resulted in a final dataset of 401 genomes. Genome assemblies were downloaded from the PubMLST database using the built-in Contig Export plugin, which enables batch retrieval of contig assemblies in FASTA format.

### 2.2 Data quality assessment and filtering

Genome assembly quality was assessed using QUality ASsessment Tool (QUAST) v5.3.0, which provides metrics including contig sizes (number of contigs, largest contig, total length), GC (%), N50, L50, and number of ambiguous bases (N) per 100 kbp (Gurevich et al., 2013). Genome-wide completeness was quantified using Benchmarking Universal Single-Copy Orthologue (BUSCO) v6.0.0 with the *acinetobacter*_odb12 lineage dataset (Manni et al., 2021b, a). In addition, completeness and contamination were assessed using CheckM2 v 1.1.0 with “specific” neural network model for completeness prediction and gradient boost model for contamination estimation (Chklovski et al., 2023). Speciator v4.0.0 implemented in PathogenWatch was used for species-level confirmation (Gladstone et al., 2020). Whole-genome Average Nucleotide Identity (ANI) was computed using FastANI v 1.34 against the reference genome “*Acinetobacter baumannii* strain ATCC 19606 chromosome, complete genome” (NCBI ID: NZ_CP045110.1) (Jain et al., 2018). Genome assemblies with less fragmentation (≤200 contigs), consistent with expected genome size range of *A. baumannii*, high completeness (≥95%), low contamination (<2%), and ANI value >95% with reference genome, were retained for downstream analyses.

### 2.3 Sequence typing and serotyping

Genomes were analyzed for sequence types (STs) and surface polysaccharide types (capsule and outer core) using PathogenWatch v23.5.2. Sequence typing was based on the housekeeping genes defined by the Pasteur and Oxford multilocus sequence typing (MLST) schemes in PubMLST database. Minimum spanning tree (MST) of the STs based on Pasteur scheme allelic profile was constructed with GrapeTree v1.5.0 using MSTree method combined with eBurst algorithm (Zhou et al., 2018). Capsular polysaccharide loci (KL) and outer core lipooligosaccharide loci (OCL) types were based on Kaptive v3.1.0 (Wyres et al., 2020; Cahill et al., 2022).

### 2.4 Virulome and resistome prediction

Virulence factor (VF) genes, antimicrobial resistance genes (ARGs), and antibacterial biocide/metal resistance genes were identified using ABRicate v1.4.0, with a minimum identity and coverage threshold of 80%. VF screening was against the virulence factor database (VFDB) (Chen et al., 2016; Liu et al., 2022). ARGs were predicted by Comprehensive Antibiotic Resistance Database (CARD) v4.0.0 search (Jia et al., 2017; Alcock et al., 2023). Antibacterial biocide/metal resistance genes were searched against BacMet v2.0 database (Pal et al., 2014).

### 2.5 Mobilome prediction

Plasmid reconstruction and replicon typing were performed using MOB-suite (MOB-recon module) v3.1.9 (Robertson and Nash, 2018; Robertson et al., 2020). Insertion sequence (IS) elements including both complete and partial sequences were predicted using ISEscan v1.7.3 (Xie and Tang, 2017). Integrons were predicted using IntegronFinder v2.0.6 with promoter and *attI* site detection enabled, together with the *--local-max* option to improve enhanced local detection. The integrons were classified into complete, In0, and clusters of *attC* sites lacking integron-integrases (CALIN), based on the content. Complete integrons contains both an integrase and *attC* site; In0 elements includes an integrase but no *attC* sites; CALIN comprises at least two *attC* sites (Néron et al., 2022). Prophage sequences were annotated using Phigaro v2.4.0 in ‘basic’ mode, where the prediction score is calculated as the product of the phage score and GC content score, and enabled reporting of phage virus orthologous groups (VOGs) for each identified region (Starikova et al., 2020; Pearl and Anbarasu, 2025).

Genomic context of predominant acquired carbapenemase genes was investigated through gene synteny analysis using Flankophile v0.2.10 (Thorn et al., 2024). Representative reference sequences corresponding to the major carbapenemase families identified in the dataset (OXA-23-like, OXA-58, and OXA-72) were used as targets. Flanking regions extending 1,000–1,500 bp upstream and downstream of each target gene were extracted and compared across genomes using the default clustering parameters implemented in Flankophile.

### 2.6 Statistical testing

Statistical analyses were performed to compare genomic features between CRAB and CSAB genomes. Categorical variables, including STs, KL types, and OCL types, were compared using the Chi-square test. Non-parametric approaches were used to test the statistical significance of differences for continuous variables. Variables such as the abundance of VFs, ARGs, carbapenemase genes, metal resistance genes, plasmid replicons, carbapenem resistance-associated IS elements, and integrons, were assessed using two-sided Mann-Whitney U test (Noel et al., 2022a). Median values and interquartile ranges (IQRs) were estimated to summarize the group-wise distributions and variations. In addition to significance testing, effect size was assessed using Cliff’s *δ* to calculate the magnitude and direction of differences between groups. Effect sizes were interpreted as negligible (<0.147), small (0.147–0.33), medium (0.33–0.474), or large (>0.474). Fisher’s exact test was used for comparative analysis of transposable prophage-associated sequences. A *p*-value < 0.05 was considered statistically significant unless otherwise specified.

### 2.7 Pangenome analysis and pangenome-wide association study

#### 2.7.1 Pangenome partitioning and core genome phylogeny construction

Structural gene feature prediction and genome annotation were performed using Prokka v1.15.6 with genus-specific BLAST databases search and minimum contig length of 200 bp (Seemann, 2014; Chandy et al., 2024; Pearl et al., 2025). The pangenome portioning and core gene alignment was performed using Panaroo v1.6.0 (Tonkin-Hill et al., 2020). Core genome-based phylogeny was constructed using IQ-TREE v3.1.1 with core alignment from Panaroo’s output (Nguyen et al., 2015). Pangenome plots were visualized using Python script from Roary.

#### 2.7.2 Pangene and antibiotic resistance association analysis

Pangenome-wide association study (PanGWAS) was performed using pyseer v1.4.0 to identify accessory genes associated with carbapenem resistance (Lees et al., 2018). Gene presence–absence matrix generated from pangenome analysis was used as input for testing carbapenem resistance association. All pangenome-derived gene clusters were initially included in the association analysis. Core genome-based phylogenetic tree generated from the core gene alignment was used to account for population structure. Pairwise phylogenetic similarity matrix was computed using the phylogeny_distance.py script implemented in pyseer. Association analysis was tested for the carbapenem resistance phenotype under a linear mixed model (LMM) framework for population structure correction. Total number of unique gene presence–absence patterns for multiple testing correction was estimated using built-in pyseer count_patterns.py script. Bonferroni correction method was applied using the identified unique patterns as threshold and significant associations were filtered using R script. Tested genes labelled as “bad-chisq” were excluded.

Subsequently, unnamed group clusters without functional annotations were filtered out from the tested associations for biological interpretations. Further, rare variants with allele frequency (AF) <0.08 were excluded to minimize unstable associations arising from sparsely distributed accessory genes in the population-structured dataset. Application of this threshold excluded only one low-frequency variant (AF = 0.03), indicating minimal impact on the overall association results. Additional filtering step included retention of entries with positive beta coefficients for downstream biological interpretation, as the primary objective was to identify accessory genes positively associated with the carbapenem-resistant phenotype. To further examine the influence of lineage structure on the identified PanGWAS associations, lineage-aware prevalence analysis was performed for the significantly associated (positive associations) accessory genes across major STs and carbapenem resistance phenotypes. Percentage gene prevalence within each lineage–phenotype group were calculated from binary presence–absence profiles using custom Python scripts and visualized as a heatmap.

### 2.8 Variant calling and core SNP phylogeny construction

Variant calling and core SNP alignment was performed with Snippy v4.6.0, with “*Acinetobacter baumannii* ACICU, complete sequence” (NCBI GenBank ID: NC_010611) as the reference for mapping. Variants were identified across all the genomes using snippy-multi script and core variant profile was compiled by snippy-core. Core full alignment was cleaned using snippy-clean_full_aln script to remove poor quality and gapped regions. Gubbins (Genealogies Unbiased By recomBinations In Nucleotide Sequences) v3.4.3 was used to detect and remove the recombinant regions in cleaned full alignment (Croucher et al., 2015). SNP-sites v2.5.1 was then used to extract SNP positions to construct a concise core SNP alignment (Page et al., 2016). A maximum-likelihood tree was built with FastTree v2.2.0 from core SNP alignment using GTR model (Price et al., 2009). Pairwise SNP distances between genomes were estimated using snp-dists v1.2.0.

## 3 Results

### 3.1 Genomic dataset and quality filtering

Genomes were systematically curated to include only *A. baumannii* genomes with clinical origin, year of isolation (≥2001), and available carbapenem susceptibility data. This stringent filtering was applied to ensure reliable genotype–phenotype correlations and comparative analyses. The curated dataset of 401 A*. baumannii* genomes were majorly derived from isolation sources namely wound, blood, lower respiratory tract, and urine samples. Of the 401 genomes, 272 were assigned as CRAB and 129 as CSAB. The overall resistant profile from metadata shown that among the 272 CRAB genomes, 51 were identified as MDR, 165 as XDR, and 42 as PDR.

Assembly quality metrics and completeness-based filtering resulted in a final dataset of 395 genomes consisting of 267 CRAB and 128 CSAB. The dataset comprised of genomes with a mean contigs of 70 per genome, with an average genome size of 4 Mbp and GC content of 39%. The mean N50 and L50 values were 2,22,484 bp and 9, respectively, while the average number of ambiguous bases was 1 N per 100 kbp. BUSCO analysis demonstrated a high level of genome completeness, with an average completeness score of 99.6% corresponding to an average of 1,480 complete BUSCOs out of 1,486 searched orthologue groups. CheckM2 analysis further confirmed genome assembly dataset quality, with mean completeness and contamination values of 100% and 0.3%, respectively. Species confirmation using Speciator (PathogenWatch) identified all retained genomes as *A. baumannii*, with no closely related *Acinetobacter* species detected in the final dataset. ANI estimation revealed a mean similarity of 97.8% relative to the reference genome. Collectively, the stringent metadata curation and multi-layered genome quality assessment ensured a final high-confidence dataset for downstream comparative genomic and association analyses.

### 3.2 Population structure

#### 3.2.1 Sequence types and clonal diversity

**Figure 1:**
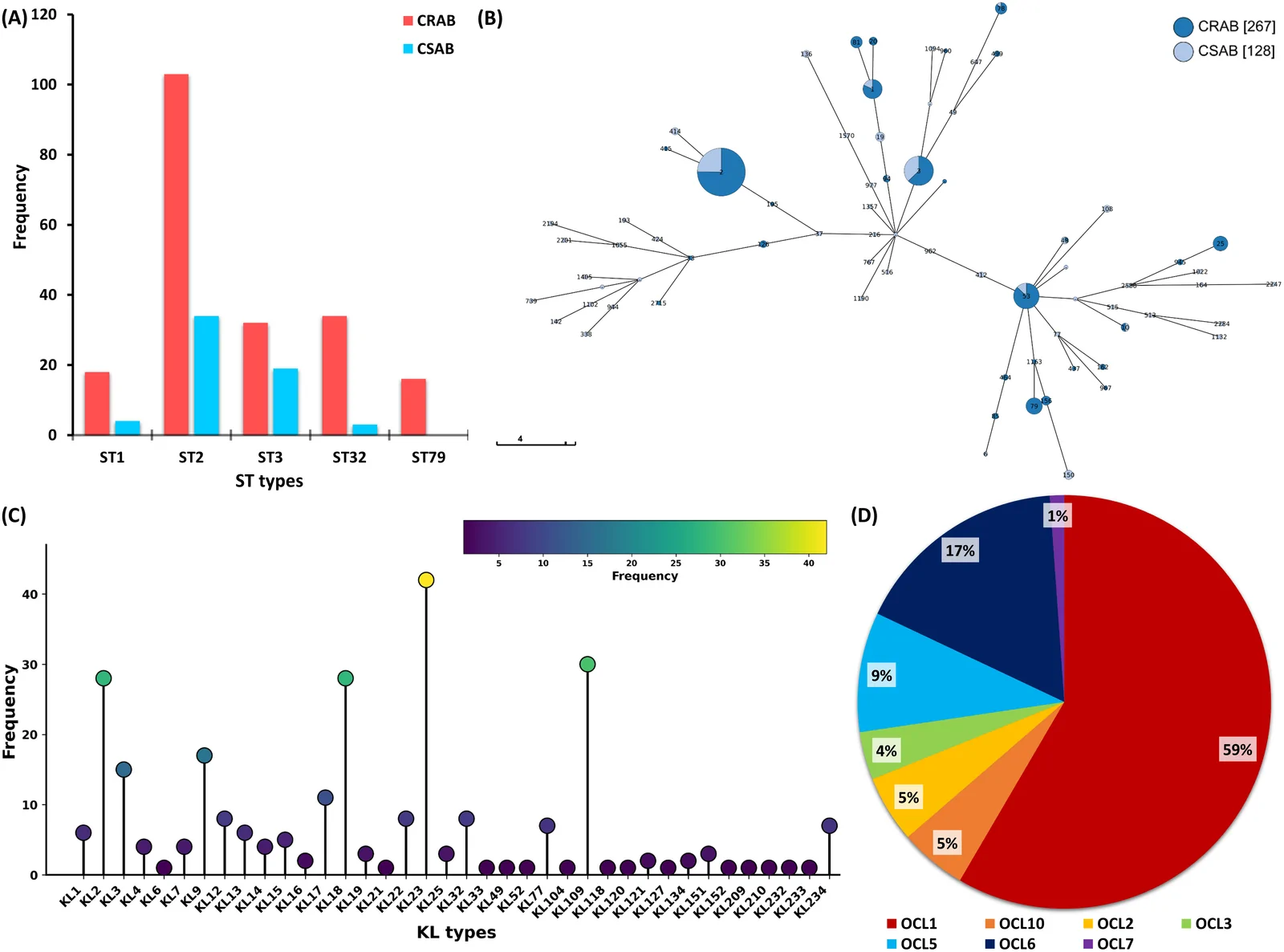
Sequence type and surface polysaccharide profiles. **(A)** Comparative distribution of the top five prevalent STs between CRAB and CSAB genomes **(B)** Minimum spanning tree based on the Pasteur MLST allelic profiles of CRAB and CSAB genomes, annotated using pie-chart nodes representing phenotype distribution **(C)** Lollipop plot showing the distribution and relative frequency of capsular polysaccharide locus (KL) types among CRAB genomes **(D)** Distribution of outer core lipooligosaccharide locus (OCL) types among CRAB genomes.

MLST revealed a highly diverse distribution of ST across both CRAB and CSAB genomes. Despite the diversity, the studied dataset was dominated by several globally recognized lineages. ST2 was the most prevalent lineage, accounting for 38.6% (n = 103) of CRAB and 26.6% (n = 34) of CSAB populations, indicating its predominance across both phenotypes. Other prevalently observed STs in CRAB included ST32 (n = 34), ST3 (n = 32), ST1 (n = 18), and ST79 (n = 16) (**Figure 1A**). CRAB genome subset comprised of 27 STs while CSAB comprised of 48 STs. A substantial number of low-frequency STs were also identified, particularly in the CSAB genomes, reflecting higher genetic heterogeneity among susceptible populations. Statistical analysis using Chi-square test for the top five predominant STs (ST2, ST32, ST3, ST1, and ST79) confirmed that the distribution of these STs differed significantly between CRAB and CSAB populations (*χ*² = 15.89, df = 4, *p* = 0.003), indicating a lineage-associated structure in carbapenem resistance. MST showing the clustering relationship of allelic profiling of STs based on Pasteur scheme is depicted in **Figure 1B**. Overall, these observations suggest that carbapenem resistance is distributed across multiple clonal groups but is disproportionately concentrated within a limited number of internationally disseminated lineages, particularly ST2 and related high-risk clones.

#### 3.2.2 Surface polysaccharide types

Capsular polysaccharide diversity determined through KL typing revealed heterogeneity across the dataset. A total of 70 different KL types were identified, with several types exhibiting varying distribution between CRAB and CSAB subsets. Among these, KL23 (n = 42), KL109 (n = 30), KL18 (n = 28), and KL2 (n = 28) were predominantly observed KL types in CRAB genomes (**Figure 1C**). Chi-square test shown significant difference in the KL distribution between CRAB and CSAB populations (*χ*² = 178.64, df = 69, *p* < 0.001).

Outer core lipooligosaccharide diversity assessed by OCL typing exhibited a conserved distribution. OCL1 was observed as the predominant type, accounting for 58.4% of CRAB and 62.5% of CSAB genomes, indicating its widespread prevalence across both populations. Other OCL types such as OCL6, OCL5, OCL10, OCL2, and OCL3 were also identified in CRAB genomes in slightly higher proportion than CSAB (**Figure 1D**). Despite the dominance of OCL1 across both groups, Chi-square test revealed that OCL distribution differed significantly between CRAB and CSAB subsets (*χ*² = 37.33, df = 12, *p* < 0.001).

The observed KL and OCL types diversity highlights substantial surface polysaccharide heterogeneity within the study population and varied profile among the CRAB and CSAB subsets.

### 3.3 Profiling of virulome

**Figure 2:**
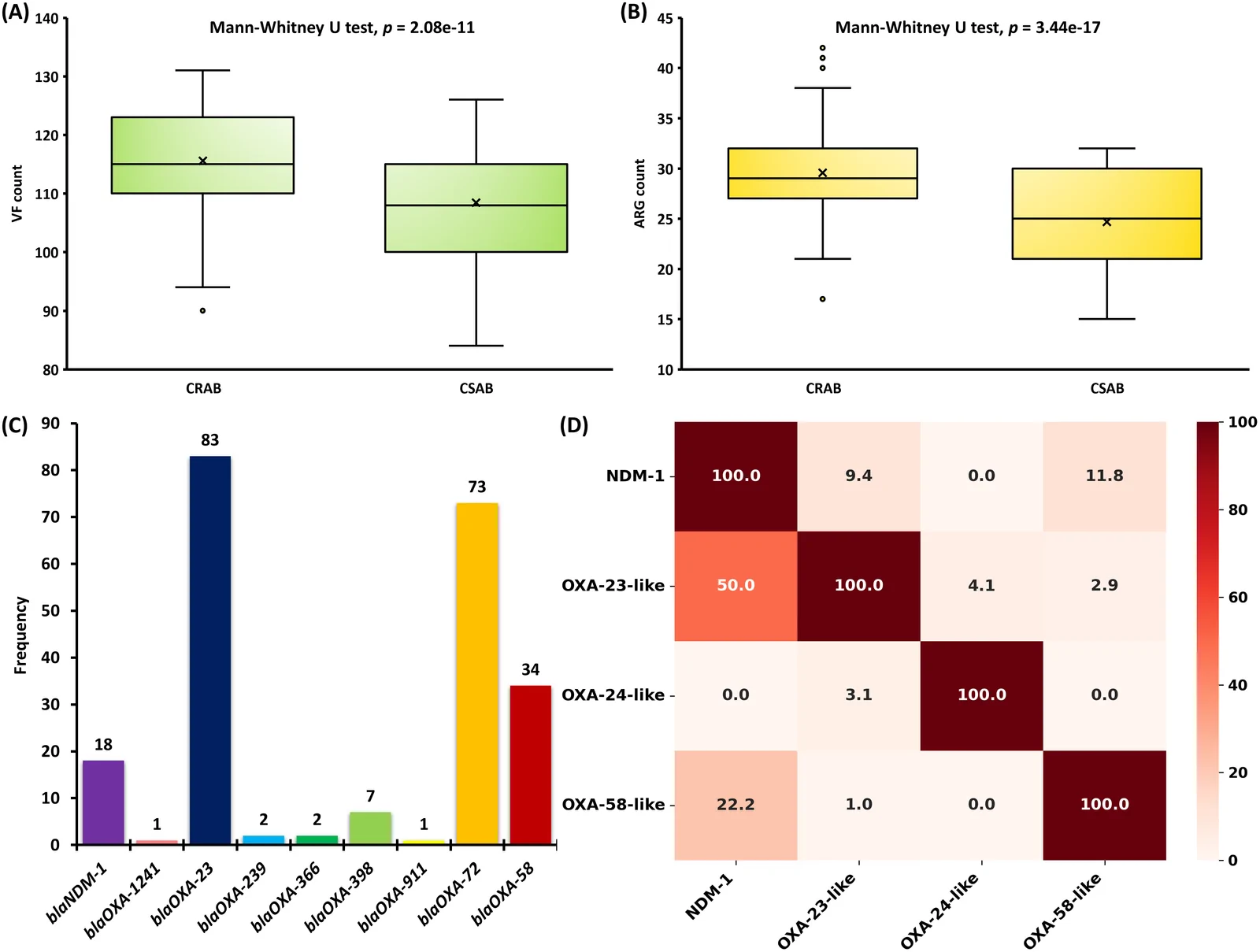
Virulome and resistome characteristics. **(A)** Box plot comparing virulence factor (VF) counts between CRAB and CSAB genomes. Boxes represent the interquartile range (IQR), the horizontal line within each box indicates the median, and whiskers represent data dispersion beyond the quartiles **(B)** Box plot comparing total antimicrobial resistance gene (ARG) counts between CRAB and CSAB genomes. Boxes represent the IQR, the horizontal line within each box indicates the median, and whiskers represent data dispersion beyond the quartiles **(C)** Frequency distribution of acquired carbapenemase families among CRAB genomes **(D)** Heatmap depicting co-occurrence patterns among major acquired carbapenemase families identified in CRAB genomes. Values represent the percentage of genomes carrying the carbapenemase family represented on the x-axis that additionally harbor the carbapenemase family represented on the y-axis.

Comparative analysis of the virulome shown a moderately higher VF burden in CRAB genomes compared to CSAB. The median number of VFs per genome was 115 (IQR = 110–123) in CRAB and 108 (IQR = 100–115) in CSAB, with a statistically significant difference (Mann-Whitney U test, *p* < 0.001), indicating a moderate effect size (Cliff’s *δ* = 0.415) (**Figure 2A**). Despite this difference, the near similarity in distribution of VF counts between the two groups suggests that virulence potential is conserved across both CRAB and CSAB.

Functional categorization of identified VFs revealed that genes associated with adherence, effector delivery systems, immune modulation, and metabolic adaptation were predominant in both the groups. In CRAB, adherence-associated genes (n = 7299), effector delivery systems (n = 6621), and immune modulation genes (n = 5509) represented the most prevalent categories. Systems such as type VI secretion system (T6SS), type II secretion system (T2SS), biofilm-associated factors, and iron acquisition systems (acinetobactin) were consistently identified across both groups. Overall, CRAB genomes showed a modest but significant burden of VFs compared with CSAB, indicating that resistance and virulence traits may co-accumulate within successful clinical lineages.

### 3.4 Profiling of biocide and metal resistome

Antibacterial biocide/metal resistance gene profile comparison revealed no significant difference in per-genome gene counts between CRAB and CSAB groups (*p* = 0.205), supported by a negligible effect size (Cliff’s *δ* = 0.076). Most genes of this category, particularly those encoding multidrug efflux pump systems such as *adeABC*, *adeFGH*, and *abeM*, were highly conserved across both subsets, suggesting the intrinsic mechanistic characteristics of these determinants. Though several disinfectant resistance determinants (*qacE*Δ1, *qacE*, *sugE*) and heavy metal resistance genes (*mer* operon components, *cadD*, *nreB*) were identified, their distribution did not differ significantly. However, these genes can potentially co-select with ARGs and can contribute to cross- or co-resistance mechanisms.

### 3.5 Profiling of antibiotic resistome

#### 3.5.1 Overall ARG burden

Comparative analysis of the resistome revealed a substantially higher ARG burden in CRAB genomes compared to CSAB genomes. The median ARG count per genome was 29 (IQR = 27–32) in CRAB genomes and 25 (IQR = 21–30) in CSAB genomes, corresponding to average values of 30 and 25, respectively. Mann-Whitney U test confirmed high statistical significance of this difference (*p* < 0.001), with a large effect size (Cliff’s *δ* = 0.522) (**Figure 2B**). These observations highlight that carbapenem resistance is augmented by a wider expansion of the resistome, underscoring the MDR characteristic of contemporary CRAB strains.

#### 3.5.2 Carbapenem resistance genes and carbapenemase distribution

Functional classification the identified ARGs revealed genes associated with carbapenem antibiotics resistance were markedly higher in CRAB genomes (total gene count = 1,568) compared to CSAB genomes (total gene count = 639). Efflux pump-associated resistance genes, particularly components of the AdeIJK system associated with carbapenem molecules efflux, were widely distributed across both CRAB and CSAB genomes. Core genes (*adeI*, *adeJ*, and *adeK*) and regulatory gene *adeN* were present in nearly all genomes. Analysis of enzymatic inactivation mechanism-associated carbapenem resistance genes shown a significant prevalence of genes encoding acquired carbapenemases in CRAB genomes, whereas these determinants were absent in CSAB. The median number of acquired carbapenemase genes per genome was 1 (IQR = 1–1) in CRAB genomes, with a high significant difference (Mann–Whitney U test, *p* < 0.001) and larger effect size (Cliff’s *δ* = 0.768), indicating a strong association with the carbapenem resistant phenotype.

Among the identified acquired carbapenemases, genes coding for OXA-type class D *β*-lactamases were predominant, including OXA-23-like (n = 96), OXA-24-like (n = 73), and OXA-58-like (n = 34) variants detected in CRAB genomes. Additionally, NDM-1 MBLs (n = 18), PER-1-like ESBLs (n = 15), GES-11 ESBL (n = 3) were also identified in CRAB genomes (**Figure 2C**). Genes encoding OXA-51-like enzymes were detected in nearly all genomes, consistent with their known role as intrinsic chromosomally encoded *β*-lactamases in *A. baumannii*. Collectively, these findings indicate that carbapenem resistance in this dataset is primarily driven by the acquisition of OXA-type carbapenemases.

#### 3.5.3 Carbapenemase co-occurrence patterns

Co-occurrence analysis revealed partial overlap between NDM-1 and OXA-23-like carbapenemases, with 50% of OXA-23-like-positive genomes additionally carrying NDM-1, whereas only 9.4% of NDM-1-positive genomes harbored OXA-23-like enzymes. In contrast, OXA-24-like and OXA-58-like carbapenemases shown limited co-occurrence with other carbapenemase families, indicating independent dissemination patterns (**Figure 2D**). The co-occurrence of multiple carbapenemase families suggests the complex resistance networks harbored within CRAB genomes.

### 3.6 Profiling of mobilome

#### 3.6.1 Plasmid reconstruction and typing

**Figure 3:**
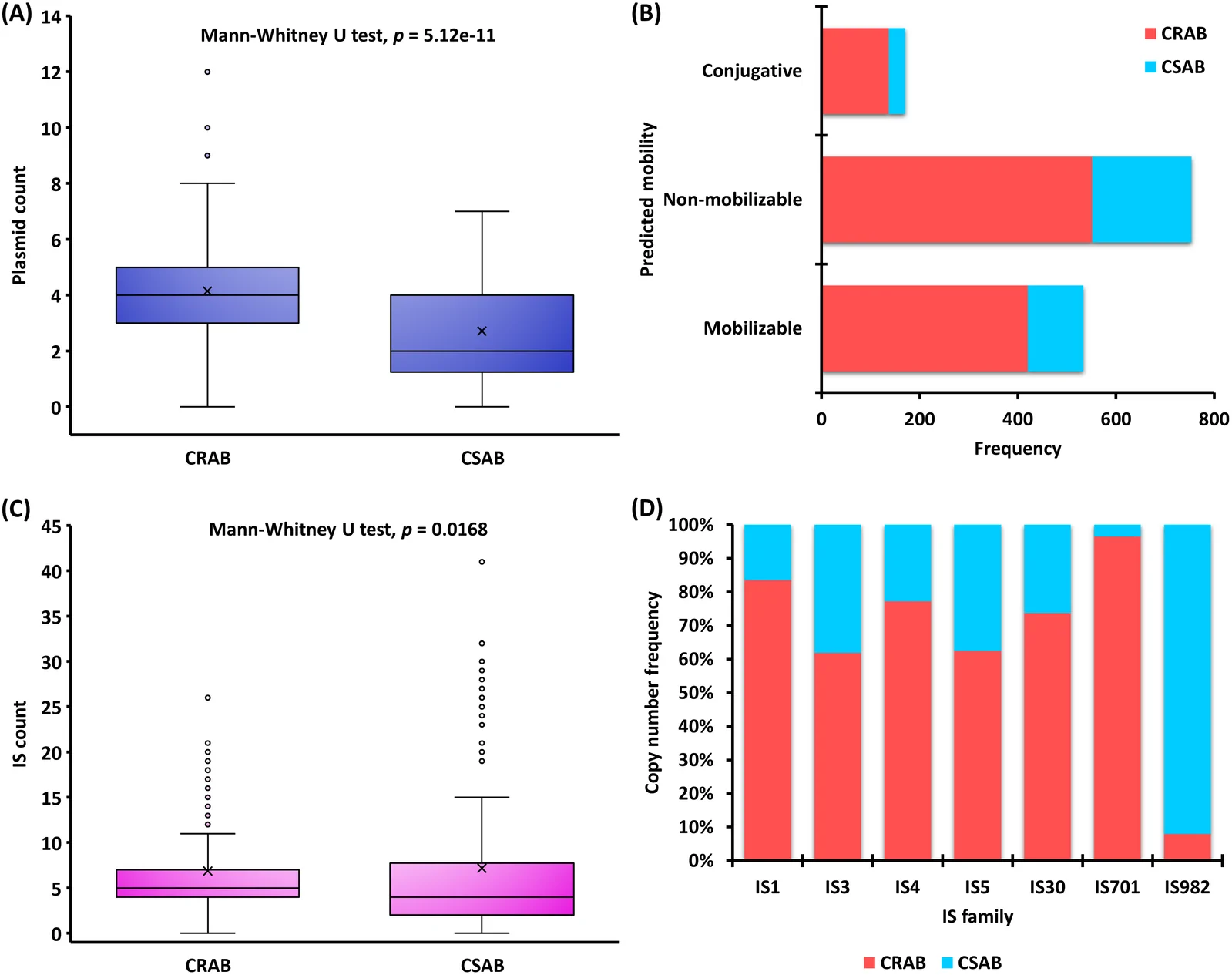
Mobilome-associated features. **(A)** Box plot comparing plasmid contig counts between CRAB and CSAB genomes. Boxes represent the interquartile range (IQR), the horizontal line within each box indicates the median, and whiskers represent data dispersion beyond the quartiles **(B)** Distribution of predicted plasmid mobility classes among CRAB and CSAB genomes **(C)** Box plot comparing carbapenem resistance-associated insertion sequence (IS) counts between CRAB and CSAB genomes. Boxes represent the IQR, the horizontal line within each box indicates the median, and whiskers represent data dispersion beyond the quartiles **(D)** Stacked bar chart showing the distribution of carbapenem resistance-associated IS families in CRAB and CSAB genomes.

Comparative analysis of plasmidome revealed a higher plasmid burden in CRAB subset (median = 4 per genome) than CSAB genomes (median = 2 per genome). Mann-Whitney U test showed significant difference (*p* < 0.0001), along with medium effect size (Cliff’s *δ* = 0.403), suggesting expanded accessory genomic content associated with plasmids in CRAB genomes (**Figure 3A**). A total of 1,108 plasmid contigs were detected among 263/267 (98.5%) CRAB genomes, whereas 349 plasmid contigs were identified among 113/128 (88.3%) CSAB genomes. Single-replicon plasmids predominated in both groups. Analysis of distribution of predicted mobility of the identified plasmid contigs shown predominance of mobilizable plasmids in both CRAB and CSAB isolates. However, CRAB genomes harbored higher conjugative plasmids (n = 137), identified in 133/267 (49.8%) genomes, compared to 33/128 (25.8%) CSAB genomes, supporting enhanced horizontal dissemination potential among carbapenem-resistant strains (**Figure 3B**). Relaxase profiling identified MOBP, MOBQ, and MOBF as the prevalent relaxase families, with MOBP-type relaxases being the most abundant across both CRAB and CSAB. Among the mating pair formation (MPF) systems, MPF_F_ type was the predominant system. Overall, these findings indicate that CRAB population possess a complex accessory genomic content and potentially transmissible plasmid repertoire that may facilitate acquisition and dissemination of resistance-associated genes.

#### 3.6.2 Insertion sequence elements

Comparison of IS element profile exhibited widespread distribution of IS elements across both CRAB and CSAB subsets. Mann-Whitney U test for carbapenem resistance-associated IS burden (IS1, IS3, IS4, IS5, IS30, IS701, and IS982) demonstrated that CRAB genomes (median = 5) exhibited a modest but statistically significant increase (*p* = 0.0168; Cliff’s *δ* = 0.148), compared to CSAB genomes (median = 4) (**Figure 3C**). IS families associated with carbapenem resistance dissemination and gene regulation were predominantly identified in CRAB genomes (**Figure 3D**). The higher occurrence of carbapenem resistance-associated IS elements in CRAB genomes indicates their role in resistance gene mobilization, expression, and genomic plasticity.

#### 3.6.3 Integrons

Integron profiling revealed presence of integron components in 76.0% of CRAB subset (n = 203) and 59.4% of CSAB genomes (n = 76). A total of 238 integrons were identified in CRAB genomes and 88 in CSAB. Among these, 143 (CRAB) and 48 (CSAB) were complete integrons. Comparative statistical analysis using Mann-Whitney U test showed a significantly higher integron burden in CRAB genomes (median = 1; IQR = 1–1) than in CSAB genomes (median = 1; IQR = 0–1) (*p* = 0.0072), although the effect size was negligible (Cliff’s *δ* = 0.143). Gene cassette profiling revealed higher diversity among CRAB integrons. Aminoglycoside resistance determinants including *aac*, *ant*, and *aadA* family genes were frequently observed in both CRAB and CSAB. However, CRAB integrons harbored additional resistance genes such as *bla*_GES_, *qnrVC*, *arr*, *catB*, *cmlA*, *dfrA*, and *sat2*, associated with resistance in *β*-lactams, fluoroquinolones, rifampicin, chloramphenicol, trimethoprim, and aminoglycosides class of antibiotics. Notably, *qacE*, a disinfectant resistance-associated element, was identified prevalently within both CRAB and CSAB integron structures. Although integrons were identified in both populations, their higher abundance among CRAB genomes indicates an increased capacity for acquisition and maintenance of accessory resistance gene cassettes for conferring resistance to multiple other classes of antibiotics.

#### 3.6.4 Prophages

Prophages were detected across both CRAB and CSAB subsets, with a total of 1,250 prophage sequence detected in CRAB isolates and 499 in CSAB isolates. CRAB genomes showed a substantially higher average prophage burden (mean = 5 prophages per genome) than CSAB (mean = 4 prophages per genome). Among the identified prophages, 31 transposable prophages were detected in CRAB genomes and 18 in CSAB. Statistical testing using Fisher’s exact test showed no statistically significant relationship between the presence of transposable prophages and carbapenem resistance phenotype (*p* = 0.516; odds ratio = 1.25), suggesting transposable prophage sequences were not associated with carbapenem resistance within the studied population.

#### 3.6.5 Genetic context of major acquired carbapenemase genes

**Figure 4:**
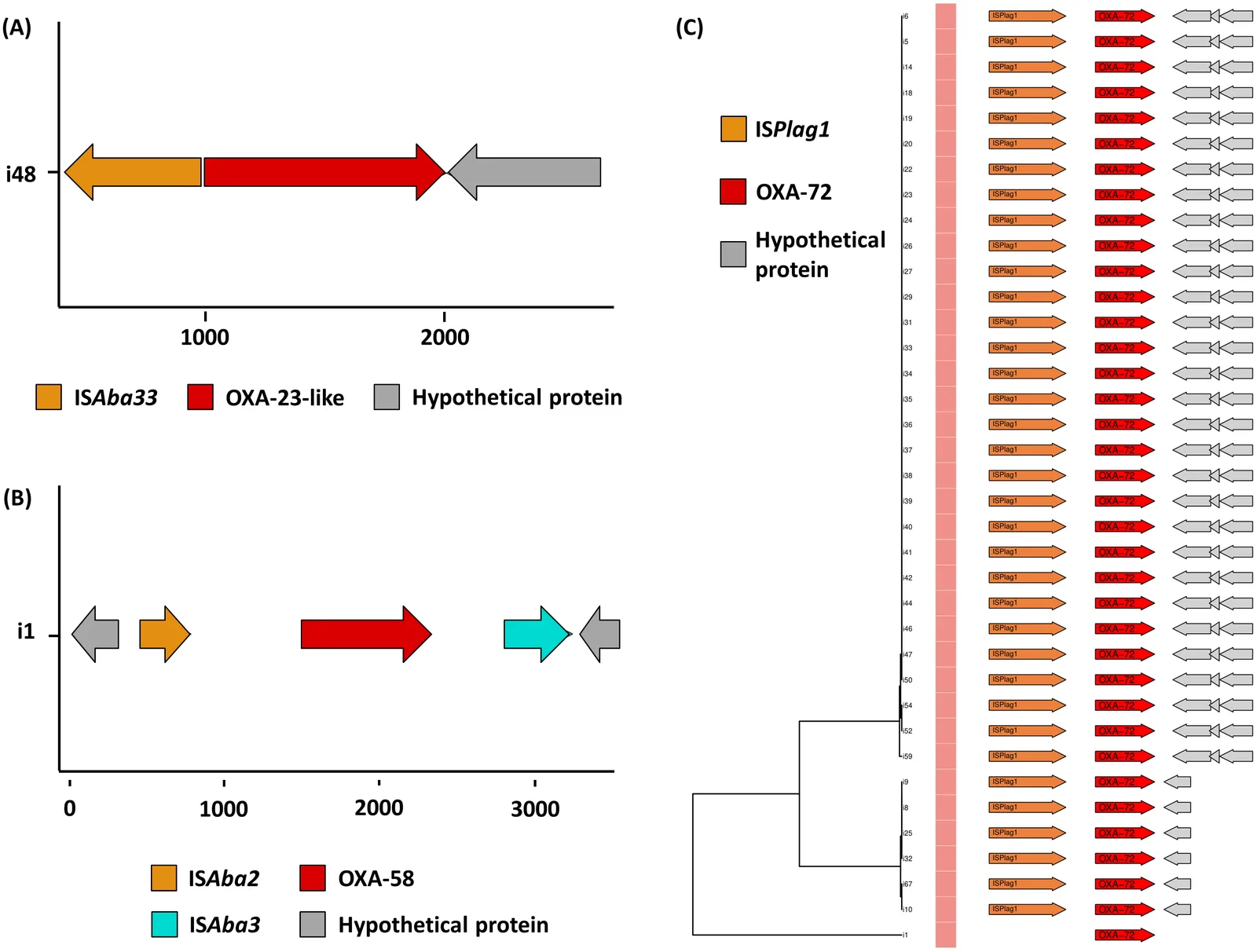
Genomic contexts of major acquired carbapenemases and insertion sequence (IS) elements co-localization **(A)** OXA-23-like **(B)** OXA-58 **(C)** OXA-72.

Investigation of the mobilization potential of acquired carbapenemase genes using gene synteny method revealed conserved flanking genomic regions surrounding the predominant carbapenemase families. OXA-23-like carbapenemases were consistently associated with neighboring IS elements, particularly IS*Aba33*, indicating a conserved mobile genetic environment. OXA-58-associated regions were characterized by the presence of IS*Aba2* and IS*Aba3* elements flanking the carbapenemase gene. OXA-72 (OXA-24-like) showed highly conserved flanking regions and were frequently linked to IS*Plag1*. Overall, these observations demonstrate that major acquired carbapenemases in the studied population occur within conserved IS-associated genomic contexts that may facilitate their mobilization and persistence among clinical *A. baumannii* lineages. Representative synteny structures are presented in **Figures 4A–C**.

### 3.7 Geographic and temporal distribution of CRAB lineages and resistance determinants

The curated genomic dataset comprised genomes originating from four continents, North America, South America, Africa, and Asia. CRAB population predominated across all continents and accounted for 69.5% of North American genomes, 54.3% of South American genomes, and all originating from Africa and Asia. Geographical differences were observed in the distribution of major STs and acquired carbapenemases. ST2 was the dominant lineage in North America (n = 126), whereas ST79 represented the most prevalent lineage in South America (n = 16). African genomes were primarily represented by ST1 (n = 8) and ST2 (n = 5). Among acquired carbapenemases, OXA-23-like enzymes were the prevalent family in Africa (100%) and South America (40.7%), while OXA-72 was most frequently identified among North American genomes (24.2%). NDM-1 was comparatively less dominant in North America but occurred at higher frequencies among African genomes (61.5%). Collectively, these observations indicate substantial regional heterogeneity in the distribution of high-risk clonal lineages and acquired carbapenemase determinants. Temporal analysis demonstrated that most genomes were collected between 2006–2010 (n = 196), followed by 2016–2020 (n = 76) and 2001–2005 (n = 68). Across all collection periods, CRAB genomes were higher than CSAB. These findings underscore the persistent global occurrence of carbapenem-resistant lineages over two decades of clinical surveillance. Despite the geographical variations in lineage composition and carbapenemase genes prevalence, predominant high-risk clones and OXA-type carbapenemases were observed across multiple regions and time periods, highlighting their global epidemiological significance.

### 3.8 Pangenome-wide association study-based accessory genome evaluation

#### 3.8.1 Pangenome structure

Genome annotation of structural gene features revealed a slightly higher genetic repertoire and coding capacity in CRAB genomes (mean = 3,864 genes; 3,800 CDS) compared with CSAB genomes (mean = 3,755 genes; 3,693 CDS). Pangenome quality control and pangenome structure partition are shown in **Supplementary Figures 2A–D**. Pangenome analysis of the 395 A*. baumannii* genomes detected a total of 13,083 gene clusters. Among these, 2,562 genes were classified as core genes, while 311 genes constituted the soft-core genome. The accessory genome was highly diverse and comprised 1,581 shell genes and 8,629 cloud genes. Core genome-based phylogeny annotated with gene presence–absence matrix is presented in **Supplementary Figure 2E**.

#### 3.8.2 Pangene and carbapenem resistance association

PanGWAS revealed several accessory genes significantly associated with the carbapenem resistance trait in the 395 A*. baumannii* genomes studied. LMM implemented in pyseer effectively corrected the population structure for phylogenetic similarity, with a heritability estimate (*h*²) of 0.88, suggesting a strong contribution of genetic background to the carbapenem resistance trait. 13,083 gene variants were screened initially, among which 6,816 low-frequency variants were pre-filtered, resulting in 6,267 tested variants. A total of 3,429 unique patterns were estimated. 285 significant associations were obtained after Bonferroni correction and downstream filtering of hits with bad Chi-square values. Subsequent removal of group clusters lacking functional annotation, low-frequency hits (AF < 0.08), and negatively associated entries resulted in six high-confidence candidate genes positively associated with the tested carbapenem resistance phenotype.

#### 3.8.3 Functional implications of positively associated significant accessory genes

**Figure 5:**
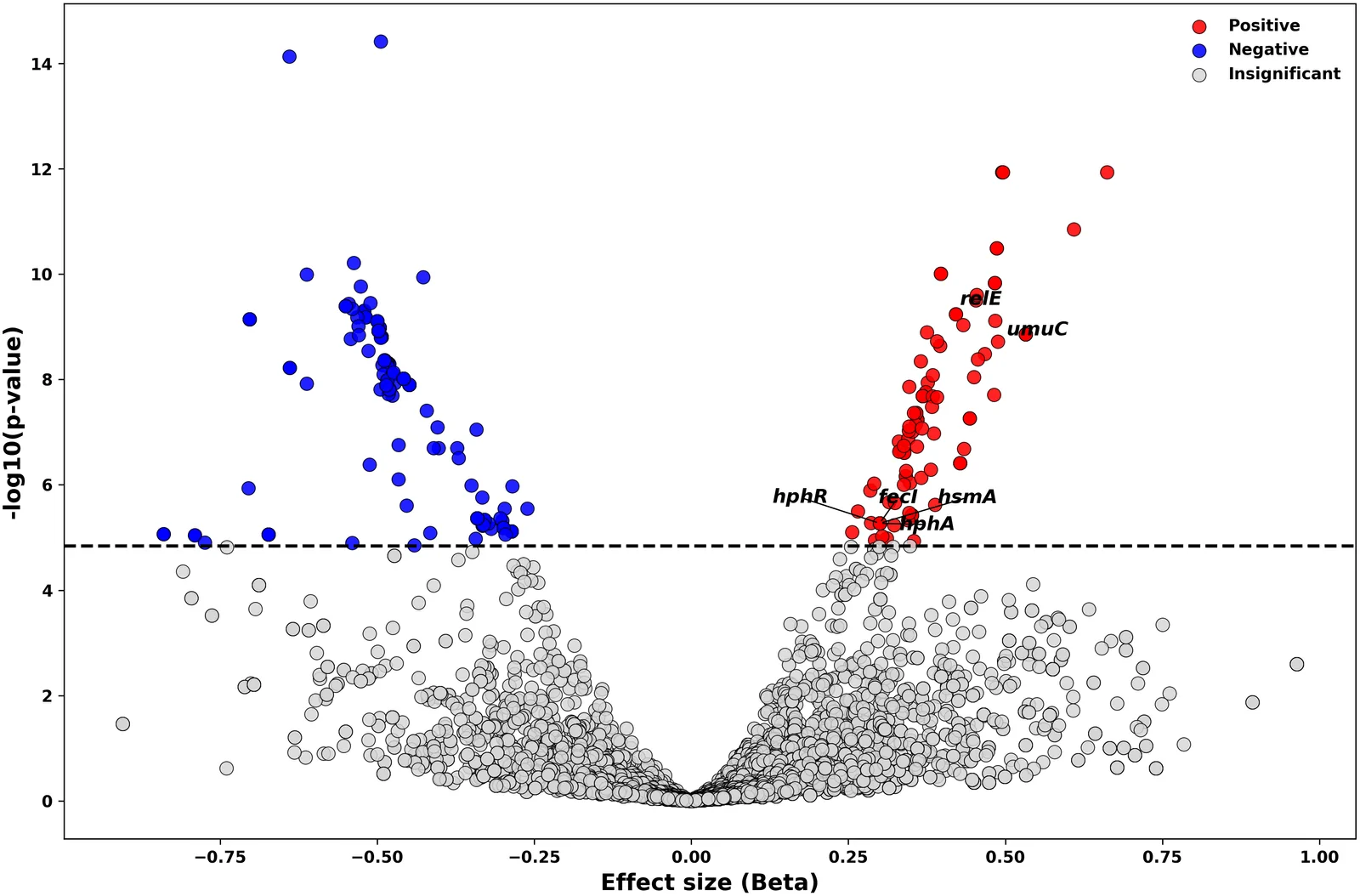
Volcano plot representing the association between accessory genes and carbapenem resistance. Magnitude of change is represented on the x-axis and statistical significance is represented on y-axis. The dashed black line shows the threshold where –log10 = 5. Positively associated genes are highlighted based on adjusted statistical thresholds and effect size estimates.

**Figure 6:**
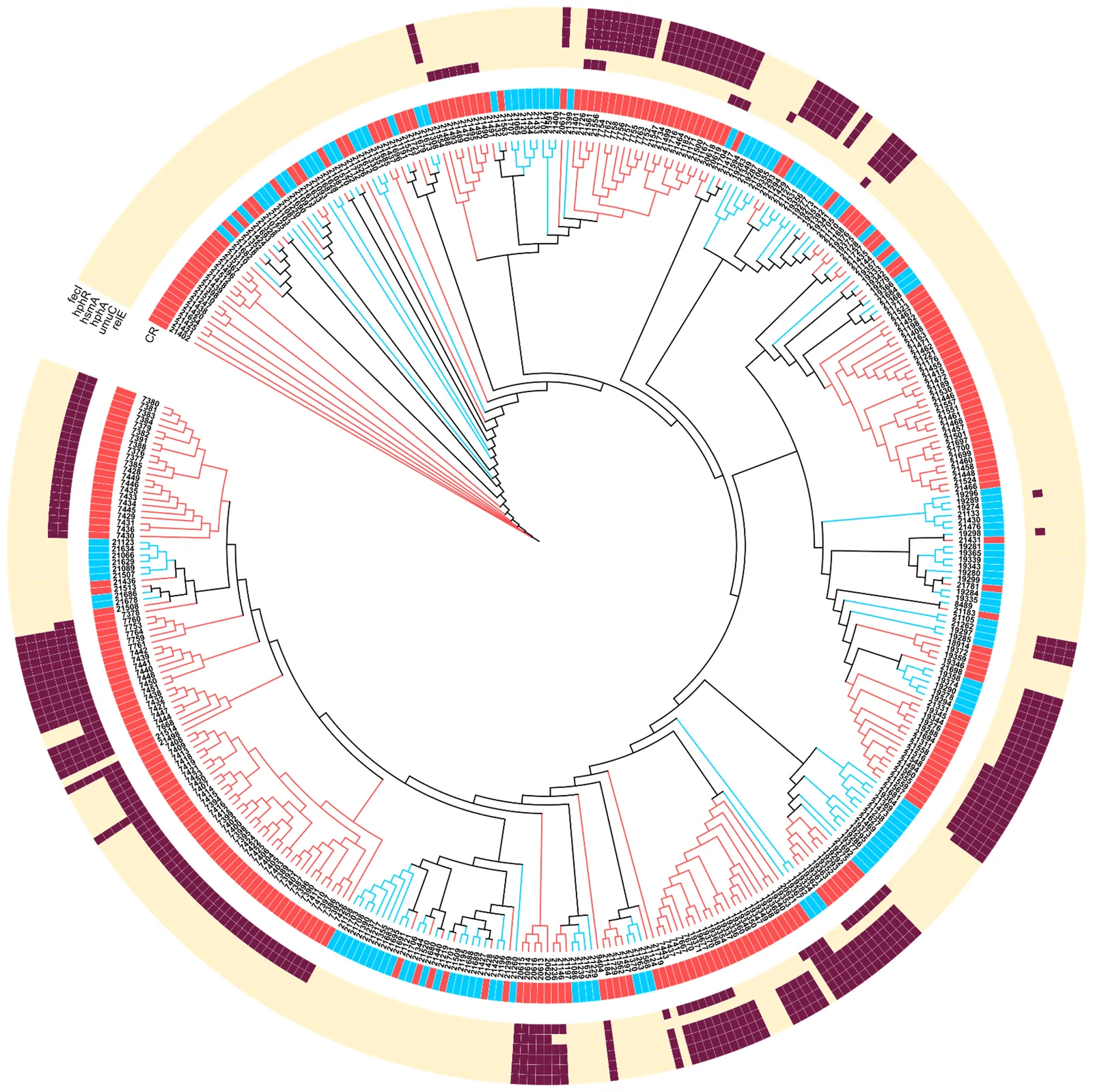
Core genome phylogeny annotated with carbapenem resistance phenotype (CRAB is denoted in red color strips while CSAB in blue) and positively associated accessory genes.

The positively associated genes included *relE*, *umuC*, *hphA*, *hsmA*, *hphR*, and *fecI* (**Table 1**). Among these, *umuC* demonstrated the highest beta coefficient (*β* = 0.488), suggesting the strongest positive association with carbapenem resistance trait. *umuC* gene codes for an error-prone translesion DNA polymerase associated with SOS-mediated DNA repair and mutagenesis. Its association with carbapenem resistance phenotype can be linked with its potential role in adaptive evolution and accumulation of resistance-associated mutations under antibiotic stress. The *relE* was observed to be substantially enriched among CRAB genomes, which encodes an mRNA interferase toxin involved in stress adaptation and bacterial persistence. This suggests its potential role in enhanced survival and persistence-mediated antibiotic tolerance. Heme acquisition-related genes such as *hphA*, *hsmA*, and *hphR* were also exhibited significant positive association with resistance phenotype. These genes constitute a high-affinity heme uptake system involved in iron acquisition and virulence. Additionally, the sigma factor gene *fecI*, which regulates ferric citrate transport, was positively associated with carbapenem resistance. A volcano plot depicting the gene associations identified through pyseer is illustrated in **Figure 5**. Overall, these genes represent the functional pathways associated with persistence, DNA damage response, and iron/heme acquisition, suggesting that successful CRAB populations may depend on adaptive traits beyond conventional direct antimicrobial resistance (AMR) mechanisms. Core genome-based phylogeny annotated with the co-occurrence patterns of the six positively associated genes is represented **Figure 6**.

**Table 1:** Significant accessory genes positively associated with carbapenem resistance phenotype identified through phylogeny lineage-aware pangenome-wide association study.

Gene	Protein	Function	Allele frequency (AF)	Filtered *p*-value	LRT *p*-value	Beta - effect size/slope of the variant	Standard error of the fit on beta	Variant *h*^2^
*relE*	mRNA interferase toxin RelE	• Toxic component of a type II toxin-antitoxin (TA) system	0.311	1.16E-17	5.75E-10	0.421	0.0662	0.305
*umuC*	Protein UmuC	• Involved in UV protection and mutation • Essential for induced (or SOS) mutagenesis	0.258	1.56E-13	1.92E-09	0.488	0.0794	0.296
*hphA*	Hemophilin	• Hemophore that acquires heme from human hemoglobin and delivers the heme to its cognate receptor, HphR, facilitating transport of heme across the bacterial outer membrane	0.289	2.07E-05	5.37E-06	0.3	0.0651	0.227
*hsmA*	Hemophilin secretion modulator	• Mediates secretion of the hemophilin HphA across outer membrane into extracellular environment	0.289	2.07E-05	5.37E-06	0.3	0.0651	0.227
*hphR*	Hemophilin receptor	• Gateway for heme entry into bacterial cell, enabling growth on hemoprotein sources • Essential for virulence, bacterial dissemination, and growth in the blood	0.289	2.07E-05	5.37E-06	0.3	0.0651	0.227
*fecI*	Ferric citrate uptake sigma factor FecI	• Initiation factors that promote the attachment of RNA polymerase to specific initiation sites and are then released • Regulates transcriptional activation of the fecABCDE operon which mediates ferric citrate transport	0.289	2.07E-05	5.37E-06	0.3	0.0651	0.227

#### 3.8.4 Population structure-aware distribution of positively associated genes

**Figure 7:**
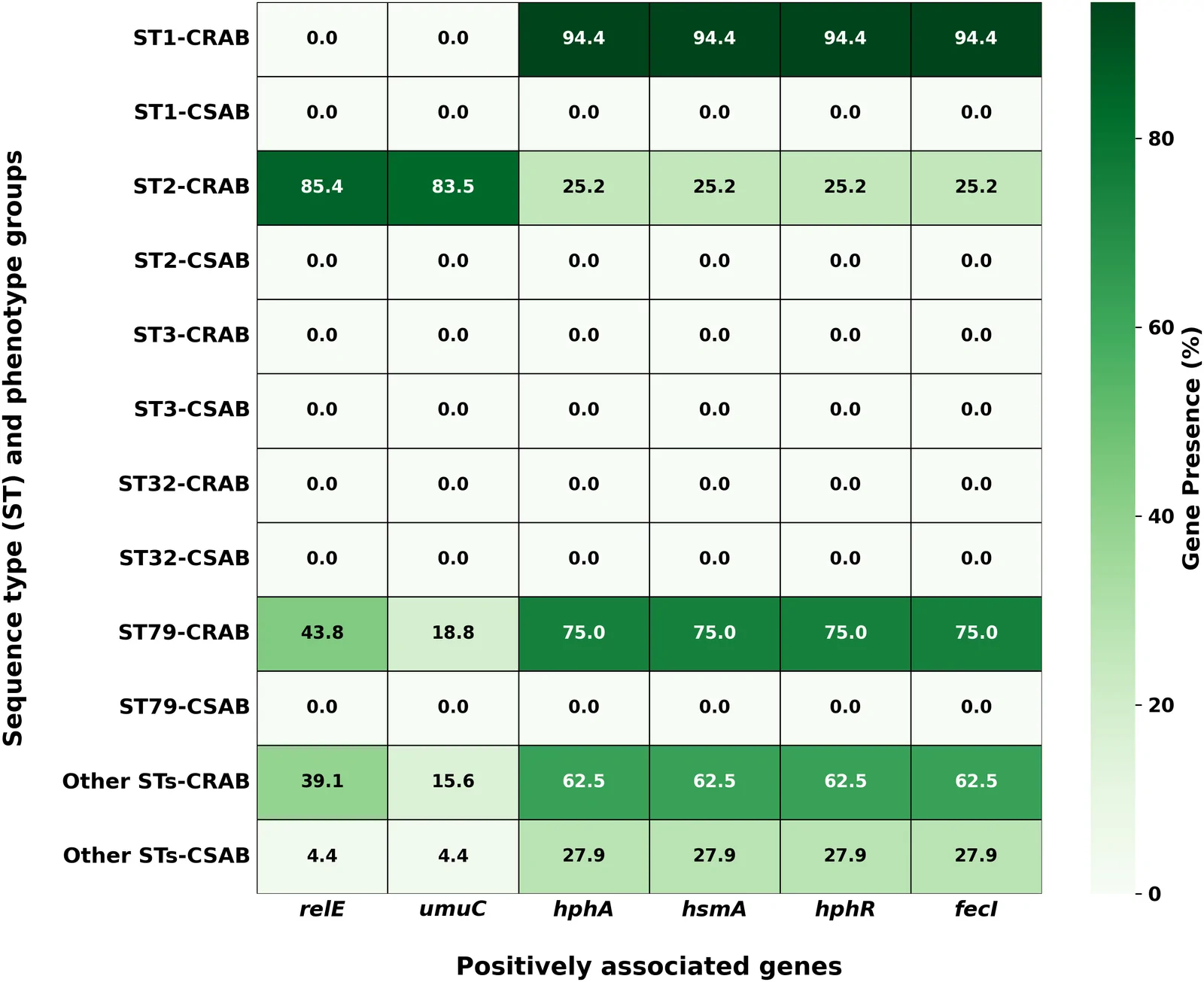
Heatmap showing lineage-based phenotype-associated prevalence of positively associated PanGWAS genes across major *A. baumannii* sequence types (STs). Values represent percentage prevalence of each gene within corresponding ST and carbapenem resistance phenotype groups. Major global lineages (ST1, ST2, ST3, ST32, and ST79) are shown individually, while remaining lineages are grouped as “Other STs”. Darker green shading indicates higher prevalence of the associated gene within the corresponding lineage–phenotype combination.

The prevalence of the six positively associated genes assessed across major STs and resistance phenotypes revealed the lineage-tested phenotype-based profiles. **Figure 7** depicts the percentage prevalence of the six significantly associated accessory genes (*relE*, *umuC*, *hphA*, *hsmA*, *hphR*, and *fecI*) across major STs (ST2, ST1, ST3, ST32, and ST79) and carbapenem resistance phenotype groups. The heterogeneous distribution patterns of these genes within dominant lineages highlights the contribution of accessory genome variation beyond population structure alone. The TA system-associated gene *relE* and DNA damage tolerance-associated gene *umuC* were predominantly enriched within ST2-CRAB genomes, occurring in 85.4% and 83.5% of genomes, respectively. In contrast, the iron acquisition-associated genes (*hphA*, *hsmA*, *hphR*, and *fecI*) shown higher occurrence in ST1-CRAB genomes, where they were present in 94.4% of genomes. These genes were additionally observed in ST79-CRAB genomes (75%) and within other CRAB-associated lineages (62.5%). Several predominant lineages, including ST3 and ST32, exhibited absence of all six genes irrespective of carbapenem susceptibility phenotype, indicating that the observed associations were not universally conserved across all CRAB lineages. Notably, the absence or substantially reduced prevalence of these genes among corresponding CSAB groups suggests that the identified positively associated genes prevalence are influenced by both carbapenem resistance phenotype and lineage-specific accessory genome composition. Overall, these findings indicate that the identified accessory genes likely represent lineage-enriched genomic signatures associated with successful resistant clades.

### 3.9 Phylogenomic analysis of ST clustering and acquired carbapenemases dissemination

**Figure 8:**
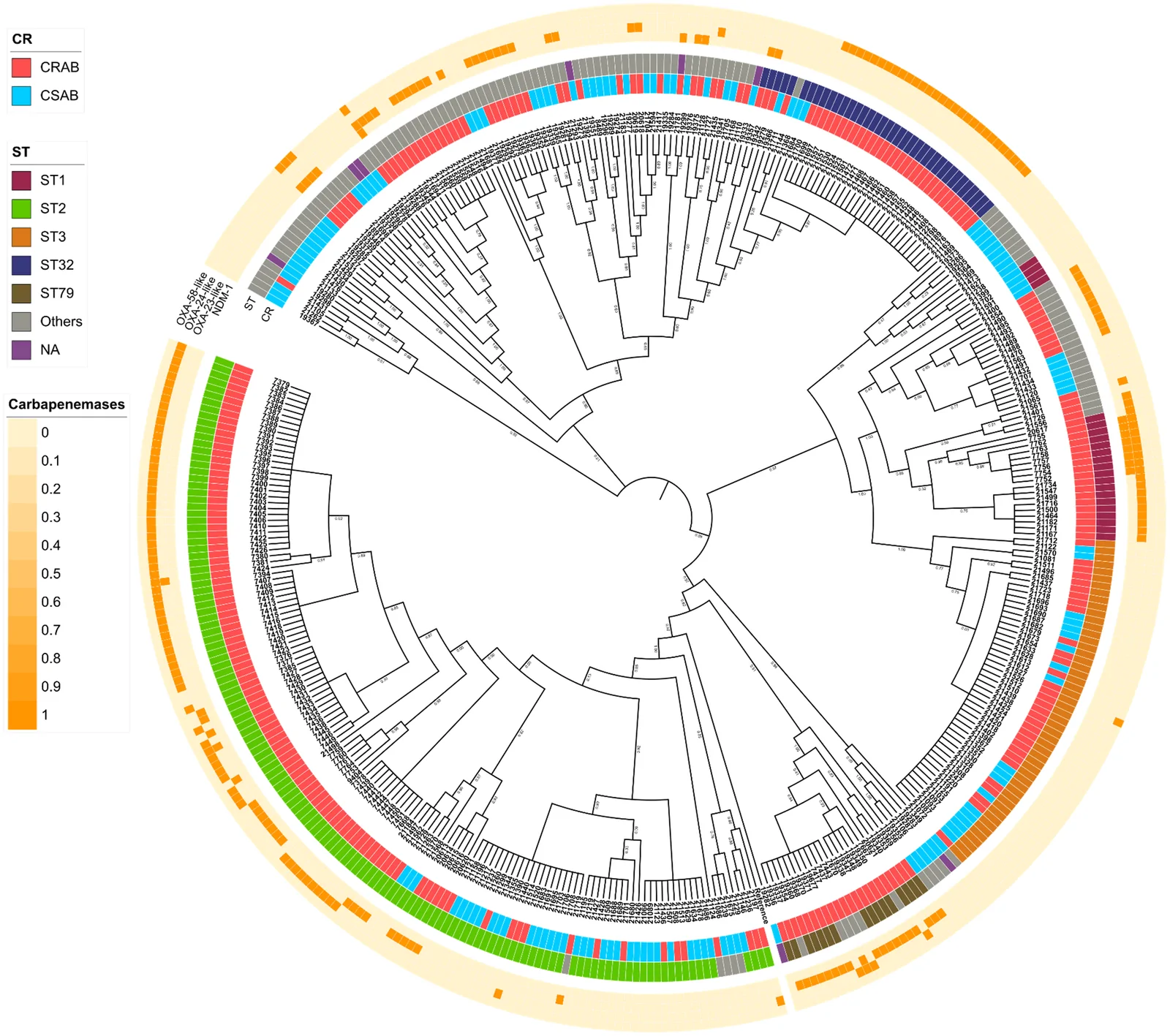
Core SNP phylogeny showing lineage distribution and acquired carbapenemase profiles.

The core SNP-based phylogeny exhibited substantial phylogenetic diversity among the analyzed 395 A*. baumannii*, with CRAB and CSAB genomes distributed across multiple evolutionary lineages (**Figure 8**). Several CRAB genomes were observed to form distinct clades dominated by globally disseminated high-risk lineages, ST2 in particular, whereas CSAB subset was more dispersed. ST1, ST3, ST32, and ST79 were also observed among multiple branches, indicating polyclonal population structure of the dataset. Annotation of the distribution of acquired carbapenemase genes showed lineage-associated enrichment patterns. Outer heatmap layer depicted heterogeneous acquired carbapenemase harboring across the phylogeny, suggesting both clonal dissemination and independent acquisition events. While several CRAB genomes formed dense resistant clusters with higher carbapenemase burden, sporadic distribution of carbapenemase-positive genomes across dispersed branches suggests the significant contribution of HGT in antibiotic resistance dissemination. Collectively, the phylogenomic architecture highlights the coexistence of clonal expansion and accessory genome plasticity in shaping the evolution of carbapenem resistance in *A. baumannii*. Pairwise SNP distances among genomes in dataset and relative to reference are shown in **Supplementary Figure 3**.

The isolate metadata, genome assembly quality metrics, AST profiles, ST and surface polysaccharide type information, VF and ARG counts, acquired carbapenemase profiles, and MGE counts are consolidated in **Supplementary Table 1**.

## 4 Discussion

CRAB strains has rapidly evolved as a formidable pathogen in the recent years, driven by its competence to persist in clinical environments and acquire diverse range of resistance determinants (Thacharodi et al., 2024). Carbapenem resistance in *A. baumannii* poses profound challenges in the treatment of infections caused by these strains. This study provides an integrated comparative perspective of the genomic determinants associated with carbapenem resistance in *A. baumannii*, highlighting that resistance is mediated through the interplay of ARGs and MGEs.

ST2 is recognized as one of the most widespread clonal lineage associated with nosocomial outbreaks, persistence, and extensive AMR. MLST analysis revealed predominance of ST2 in the studied CRAB genomes. Notably, the distribution of STs among CRAB and CSAB populations showed association of predominant STs (ST1, ST3, ST32, and ST79) alongside ST2 with CRAB genomes, while susceptible genomes exhibited higher clonal diversity and heterogeneity with no dominant lineage.

The higher ARG burden in the studied CRAB population compared to the CSAB, suggests that CRAB genomes possess an expanded resistome repertoire, consistent with the association of MDR, XDR, and PDR phenotypes with carbapenem resistance. A large-scale genomic study investigating global diversity of *β*-lactamase alleles and population structure in *A. baumannii* highlighted that the most prevalent observed among the acquired *β*-lactamase allele was *bla*_OXA-23_ (Mack et al., 2025). A Chinese study conducted in Shanghai hospitals have reported the emergence of PDR CRAB strains isolated from ICU environments, with co-harboring of OXA-23, OXA-51-like, and NDM-1 carbapenemases (Wang et al., 2025). In another study, NDM-1- and OXA-23-co-producing *A. baumannii* strain has been identified in a Spanish burn unit linked to a Libyan patient, highlighting the risk of cross-border spread of high-risk MDR clones (Hidalgo et al., 2025). Consistent with these global observations, the present study also identified *bla*_OXA-23-like_ to be the predominant acquired carbapenemase in the resistant genomes, along with the presence of *bla*_OXA-58-like_, *bla*_OXA-24-like_, and *bla*_NDM-1_ in a substantial subset of genomes (Ejaz et al., 2021; Sánchez-Urtaza et al., 2023). A subset of CRAB genomes exhibited co-occurrence of genes encoding NDM-1 and OXA-23-like carbapenemases. Co-occurrence analysis revealed that OXA-23-like carbapenemases exhibited partial co-occurrence with NDM-1, whereas OXA-24-like and OXA-58-like carbapenemases showed limited overlap with the other carbapenemase families. These findings suggest that carbapenem resistance in *A. baumannii* is majorly associated with acquisition of specific carbapenemase genes, particularly OXA-type enzymes.

The high abundance of detected plasmid contigs among CRAB genomes and the presence of mobilizable and conjugative plasmids, suggests that plasmid-mediated HGT may contribute to the dissemination of AMR determinants, particularly in clinical *A. baumannii* populations. IS4 family elements which includes IS*Aba1*, were reported to be linked to promoter activation of *bla*_OXA-23_, *bla*_OXA-51_, and *bla*_ADC_ (Saranathan et al., 2017). IS*Aba3*-driven overexpression of *bla*_OXA-58_ in *A. baumannii* isolates from hospitals in southern Vietnam has been shown to confer increased resistance to imipenem (Nguyen et al., 2020). Similarly, a WGS-based study on *Acinetobacter* spp. plasmids revealed that IS*Aba125* of the IS30 family, facilitates mobilization and Tn*125* composite transposon formation for *bla*_NDM-1_ (Alattraqchi et al., 2021). In corroboration with these previous studies, IS families (IS1, IS3, IS4, IS5, IS30, IS701, and IS982) aiding in carbapenem resistance, including members associated with the mobilization or upstream activation of carbapenemase genes such as *bla*_OXA-23_, *bla*_OXA-58_, and *bla*_NDM-1_, were widely present in the CRAB genomes of the dataset (Noel et al., 2022b). Furthermore, co-localization analysis through genomic context examination of the major acquired carbapenemase genes further supported the contribution of MGEs to carbapenem resistance dissemination. Conserved associations of OXA-23-like, OXA-24-like, and OXA-58 carbapenemases with neighboring IS elements, including IS*Aba33*, IS*Aba2*, and IS*Aba3*, highlight that these elements may contribute to gene mobilization and regulation of gene expression. Collectively, the presence of diverse MGEs underscores the extensive genomic plasticity of the studied genomes and their potential contribution in aiding the acquisition and dissemination of AMR determinants.

Pangenome analysis exhibited a highly diverse accessory genome characterized by a large proportion of cloud genes. This emphasizes the expanding genomic plasticity and open pangenome window in *A. baumannii* (Rodrigues et al., 2021). The dynamicity of *A. baumannii* pangenome demonstrates sustained evolutionary changes in resistant strains to rapidly adapt under selective pressure of antibiotics.

PanGWAS identified six positively associated genes significantly with the carbapenem-resistant phenotype, including *relE*, *umuC*, *hphA*, *hsmA*, *hphR*, and *fecI*. The TA system component *relE* has previously been associated with bacterial persistence, stress tolerance, and survival under antibiotic exposure, potentially enhancing adaptation during antimicrobial stress conditions (Keren et al., 2004). Similarly, *umuC*, encoding an error-prone DNA polymerase involved in the SOS response, contribute to increased mutational adaptability and genome plasticity under selective antibiotic pressure (Norton et al., 2013). The heme acquisition system-associated genes *hphA*, *hsmA*, and *hphR* are involved in iron utilization pathways, which are important for survival in host-associated environments and may indirectly enhance persistence and fitness of resistant clones. Similarly, *fecI*, encoding an iron uptake regulatory sigma factor, may contribute to adaptation under iron-limited host conditions. The lineage-aware prevalence analysis further highlighted that several positively associated genes were enriched within dominant CRAB-associated STs, particularly ST2, suggesting partial lineage dependence of the observed associations. *relE* and *umuC* exhibited higher occurrence within dominant CRAB-associated lineages, particularly ST2, whereas *hphA*, *hsmA*, *hphR*, and *fecI* demonstrated a more heterogeneous distribution pattern. Notably, several genomes belonging to the same ST lacked these genes, indicating that the observed associations were not exclusively lineage-defining markers but reflected accessory genome variability within phylogenetically related isolates. This heterogeneous distribution indicates that the identified genes are unlikely to represent purely lineage-defining markers and instead may reflect accessory genome acquisition, gene loss, or co-selection processes associated with carbapenem-resistant populations. Positive associations with the tested carbapenem resistance trait highlight their potential contribution to stress adaptation, persistence, virulence, and resistance-associated fitness, particularly under increased antibiotic selection pressure. Although the present study primarily focused on genes positively associated with carbapenem resistance, negatively associated accessory genes may represent susceptibility-associated genomic traits and merit further investigation.

Core SNP-based phylogenetic structure exhibited substantial genomic diversity while also demonstrating clustering patterns associated with carbapenem resistance phenotypes, major STs, and acquired carbapenemase genes. Carbapenemase gene distribution across the phylogeny showed widespread but heterogeneous dissemination of resistance determinants among CRAB lineages. Certain clades exhibited dense accumulation of carbapenemase genes, consistent with extensive acquisition and maintenance of resistance-associated accessory genomic elements. Collectively, the phylogenetic reconstruction supports the contribution of both clonal expansion and HGT to the evolution and dissemination of carbapenem resistance in *A. baumannii*.

The observed diversification and persistence of resistance determinants in *A. baumannii* underscores the genomic adaptability and highlights the need for novel therapeutic strategies to efficiently manage infections particularly in clinical settings (Naha et al., 2021; Findlay et al., 2022; Naha and Ramaiah, 2024; George et al., 2025, 2026b, a). Furthermore, stewardship strategies must balance antimicrobial administration with the AST patterns exhibited by these rapidly evolving pathogenic organisms.

This study has few limitations to be acknowledged. The reliance on draft genome assemblies may restrict complete reconstruction of plasmid architecture and precise localization of MGEs such as IS and integrons. Although statistical approaches suitable to unequal group sizes were employed, the comparatively higher proportion of CRAB genomes relative to CSAB subset may still influence certain comparative enrichment patterns and should be considered during interpretation of the findings. Additionally, the observed pangene-antibiotic resistance associations should be interpreted cautiously, as these genes may represent indirect accessory genomic signatures rather than direct carbapenem resistance determinants. Functional validation approaches including transcriptomic and proteomic profiling, targeted mutagenesis, gene knockout studies, and expression-based characterization would be necessary to confirm their accurate biological contribution to carbapenem resistance, stress adaptation, persistence, or survival under antimicrobial pressure in *A. baumannii*. Despite these limitations, the present study provides a comprehensive genomic investigation of CRAB integrating comparative genomics, PanGWAS, and phylogenomics. The identified genomic signatures and resistance-associated accessory genes improve current understanding of CRAB evolution and adaptation and may support future genomic surveillance strategies, diagnostic marker discovery, and therapeutic target exploration.

Though several findings of the present study, including the predominance of international clone lineages, enrichment of acquired carbapenemases, and higher burden of MGEs among CRAB genomes, are consistent with previous reports, the principal contribution of this study lies in the integration of high-confidence phenotypic data with large-scale comparative genomics. Beyond confirming established resistance determinants, PanGWAS identified accessory genes involved in TA systems, DNA damage response, and iron/heme acquisition that were significantly associated with carbapenem resistance phenotype. These genes are not conventional antibiotic resistance determinants and may instead represent adaptive traits that facilitate persistence and survival of successful CRAB lineages under selective pressure of antimicrobials. Further, lineage-aware analysis exhibited that although some associations were enriched within predominant high-risk international clones, the observed distribution patterns were not entirely explained by ST alone, indicating a complex interaction between genetic lineage background and accessory genome composition. Overall, these findings underscore additional biological context regarding the adaptive genomic features augmenting carbapenem resistance beyond the acquisition of carbapenemase genes themselves.

## 5 Conclusion

CRAB strains have emerged as a serious global health concern in recent years due to its ability to acquire carbapenemases *via* various HGT mechanisms, extensive presence of MGEs, and accumulation of chromosomal mutations. This study emphasizes the intricate genomic determinants associated with carbapenem resistance in *A. baumannii* genomes. The integration of population, resistome, and mobilome analyses, with comparative genomics, PanGWAS, and phylogenomics approaches provided a comprehensive view of resistance dynamics in CRAB. Carbapenem-resistant clinical *A. baumannii* genomes harbor distinct combination of acquired *β*-lactamases and MGEs compared with CSAB genomes. The predominance of high-risk clonal lineages, particularly ST2, together with the heterogeneous phylogenetic distribution of acquired carbapenemase genes, underscores the combined contribution of clonal dissemination and HGT in AMR evolution. Overall, the findings expand understanding of the genomic landscape underlying carbapenem resistance in *A. baumannii*. The identified candidate genes and accessory genomic signatures warrant further experimental validation to elucidate their mechanistic contribution to antibiotic resistance, persistence, and pathogenicity. Future research should focus on continuous monitoring and surveillance of genomic epidemiology, and clinical translations to inform antibiotic stewardship.

## Data Availability

The original contributions presented in the study are included in the article/**Supplementary Material**. Further inquiries can be directed to the corresponding author.
